# Controversies of the Effect of Ketamine on Cognition

**DOI:** 10.3389/fpsyt.2016.00047

**Published:** 2016-03-29

**Authors:** Melvyn W. B. Zhang, Roger C. M. Ho

**Affiliations:** ^1^Biomedical Institute of Global Healthcare Research and Technology (BIGHEART), National University of Singapore, Singapore; ^2^Department of Psychological Medicine, Yong Loo Lin School of Medicine, National University of Singapore, Singapore

**Keywords:** ketamine, antidepressant, cognition, memory, depression

Over the last couple of years, there have been numerous studies investigating the efficacy of ketamine, a non-competitive *N*-methyl-d-aspartic acid (NMDA) receptor antagonist as a rapid antidepressant. Most of the existing published literature describes the purported effectiveness of ketamine as a rapid antidepressant. The clinical efficacy of intravenous ketamine in the reduction of suicidal ideations ranged between that of 29% to as much as 79%. Researchers have noted the transient nature of intravenous ketamine’s rapid antidepressant efficacy and hence has recommended for long-term continued administration (1, 2). Clearly, with long-term utilization of ketamine, there are safety concerns and adverse effects that need to be considered. Prior systematic reviews have highlighted the acute adverse effects that could potential arise from the administration of intravenous ketamine (3). These adverse effects include that of dissociation, perceptual abnormalities, confusion, and transient instability in vital signs (3). Although studies have shown that serial infusions of ketamine in the short term (of 4 weeks) does not result in cognitive impairments (4), other studies have demonstrated that the administration of ketamine chronically would lead to memory impairments (5). More recently, Morgan et al. (5) in their recent article highlighted not only the increasing incidence of ketamine abuse globally but have also highlighted the mechanisms and implications of ketamine have on spatial memory. According to their study of 11 participants (of which 1 participants have had another mental health condition) who have been using ketamine recreationally, they discovered, through functional imaging studies that ketamine abusers experience spatial memory disturbances largely due to decreased activation in their right hippocampus and left parahippocampus gyrus (5). Morgan et al. (5) in their imaging studies also highlighted that chronic ketamine abusers do tend to experience dissociative symptoms, which are accounted for due to increased hippocampal activation. In addition, the functional studies also revealed that there are underlying disruption in the medial temporal lobe functioning. These resultant disruptions might also result in the psychotic symptoms that long-term ketamine abusers might experience. Clearly, the findings presented by Morgan et al. (5) are concerning if intravenous ketamine is being approved clinically and depressed individuals need to be receiving continuous administrations of intravenous ketamine. Although ketamine might seem like a promising antidepressant that could relieve treatment refractory depressive symptoms, the induction of memory impairments in the longer term is of concern. Memory impairments would result in further disabilities and consequential reduction in the overall quality of life. Aside to ketamine’s potential to result in memory impairments if used chronically, the chronic usage of ketamine does result in urological problems, such as cystitis (6).

Although it is commonly assumed that acute and chronic usage of ketamine would have deleterious effect on cognition, researchers who have looked at the efficacy of sublingual ketamine, which despite its inherent lower bioavailability of 30%, have highlighted that sublingual variant of ketamine might helped to mitigate against the cognitive adverse effects (7). In a recent trial, 77% of patients demonstrated not only improvement in their mood but also their cognition (7). Lee et al. (8) in their recent systematic review proposed the procognitive efficacy of intravenous ketamine. Lee et al. (8) recent findings raises much discrepancies and controversies with the previous established findings of ketamine on cognition. Based on Lee et al. (8) systematic review, it has been shown in studies that acute administration of low dosages of ketamine not only did not affect memory but also helped in terms of improving visual memory, simple working memory, and complex working memory in individuals with treatment-resistant depression. It has been proposed that acutely, at low doses, ketamine does have procognitive efficacy through its mechanism of action on the intracellular proteins, such as BDNF and mTORC1. Through working on these intracellular proteins, executive functioning is also affected (Figure 1). Lee et al. (8) proposed that ketamine’s acute effect on cognition might be the mediating factor that helps in the acute reduction of depressive symptoms and hence, the acute relief of suicidal ideations. It is postulated that suicidal ideations and planning for a suicide act involves much executive functioning capability. Usually, dysfunctional executive control would lead to poor impulse control and hence disinhibition. Thus, ketamine, if it does have procognitive effects could help improve the cognitive dysfunctions that usually accompanies treatment-resistant depression, as well as reduce the degree of acute suicidal ideations, through the enhancements of better executive functioning and control.

**Figure 1:**
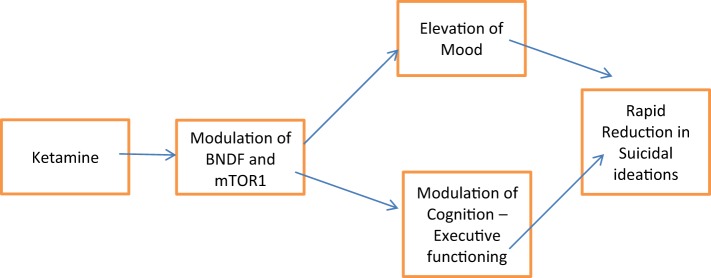
**Overview of ketamine and its precognitive effects**.

Much of the current studies have focused on investigating the efficacy of ketamine as a rapid antidepressant. Given the concerns about the adverse effects that might arise from the repeated administration, there has been a drive toward evaluating the potential of racemic mixture of ketamine. More recent studies have started to determine if the clinical efficacy of intravenous ketamine is comparable to that of conventional antidepressant, and whether ketamine could be used as an added augmentation strategy. Given the recent controversy about the commonly believed adverse effects of ketamine on cognition, this is certainly an area that deserves more research work to look into. In order to better understand whether ketamine has truly a procognitive effect, it would be worthwhile to compare sublingual or racemic variant of ketamine against antidepressants, such as Vortioxetine (9, 10), which have been hypothesized to have procognitive effects through its modulation of the 5HT7 and the 5HT3 receptors. Comparison of the racemic variant [R(−) variant of ketamine] would be key, given that prior studies have highlighted how the R(−) variant have had inherent greater potency and longer acting antidepressant efficacy (11).

## Author Contributions

All authors have contributed equally to this manuscript.

## Conflict of Interest Statement

The authors declare that the research was conducted in the absence of any commercial or financial relationships that could be construed as a potential conflict of interest.
